# Recent Progress in Micro- and Nanotechnology-Enabled Sensors for Biomedical and Environmental Challenges

**DOI:** 10.3390/s23125406

**Published:** 2023-06-07

**Authors:** Francisco J. Tovar-Lopez

**Affiliations:** School of Engineering, RMIT University, La Trobe Street, Melbourne, VIC 3004, Australia; francisco.tovarlopez@rmit.edu.au

**Keywords:** microtechnology, nanotechnology, sensors, biomedical, environmental

## Abstract

Micro- and nanotechnology-enabled sensors have made remarkable advancements in the fields of biomedicine and the environment, enabling the sensitive and selective detection and quantification of diverse analytes. In biomedicine, these sensors have facilitated disease diagnosis, drug discovery, and point-of-care devices. In environmental monitoring, they have played a crucial role in assessing air, water, and soil quality, as well as ensured food safety. Despite notable progress, numerous challenges persist. This review article addresses recent developments in micro- and nanotechnology-enabled sensors for biomedical and environmental challenges, focusing on enhancing basic sensing techniques through micro/nanotechnology. Additionally, it explores the applications of these sensors in addressing current challenges in both biomedical and environmental domains. The article concludes by emphasizing the need for further research to expand the detection capabilities of sensors/devices, enhance sensitivity and selectivity, integrate wireless communication and energy-harvesting technologies, and optimize sample preparation, material selection, and automated components for sensor design, fabrication, and characterization.

## 1. Introduction

The development of micro- and nanotechnology-enabled sensors has been a significant technological advancement that has led to several new applications in the biomedical and environmental fields [1,2,3,4,5,6,7,8]. These devices have enabled the detection and quantification of various analytes with high sensitivity and selectivity, making them ideal for applications such as vital sign monitoring [3,9,10], disease diagnosis [11,12,13], environmental monitoring [5,6,14,15,16], and food safety [17,18,19,20]. In recent years, there has been significant progress in the development of sensors, driven by the need to address critical challenges in these fields.

In the biomedical field, these devices have enabled the detection and quantification of biological molecules such as proteins and nucleic acids with high accuracy and in real time [21,22,23,24,25,26,27,28,29,30]. They have also enabled the development of point-of-care devices that can rapidly diagnose diseases and monitor treatment outcomes [31,32,33,34,35]. The use of micro- and nanotechnology-enabled sensors in the field of drug discovery and antibiotic resistance has also been significant. These devices have enabled the high-throughput screening of drug candidates and the characterization of their biological activities [4,36,37,38,39,40,41]. Additionally, the integration of sensors with emerging technologies such as artificial intelligence and machine learning has further expanded their potential applications in the biomedical field [10,42].

In the environmental field, sensors have been used to monitor the quality of air, water, and soil [6,43,44]. These devices can detect pollutants with high accuracy and in real time, enabling timely interventions to mitigate environmental pollution [16,45,46]. The use of sensors in the agricultural industry has also been significant [7,47,48,49]. These devices can monitor the quality of food and agricultural products, ensuring their safety and quality [7,48,49]. The development of biosensors that use biological components to detect environmental pollutants has further expanded their applications in this field [14,50].

**Figure 1:**
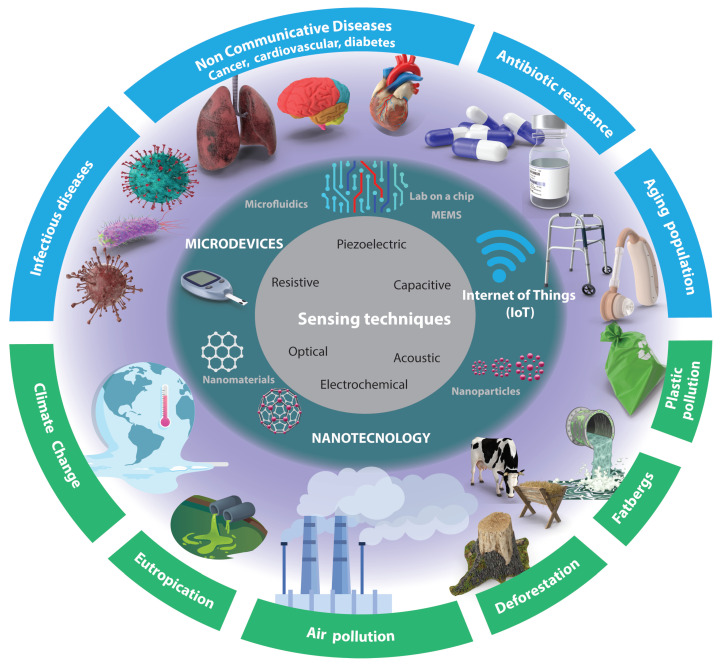
Schematic illustrating various sensing techniques and micro/nanotechnology approaches that enhance their performance in terms of specificity and sensitivity, enabling the development of new technologies to address upcoming biomedical and environmental challenges. The **innermost circle** shows fundamental sensing techniques, such as resistive sensing, capacitive sensing, piezoelectric sensing, optical sensing, acoustic sensing, and electrochemical sensing. The **middle circle** represents reported micro- and nanotechnology advances, including nanoparticles, nanomaterials, microfluidics, and MEMS. These technologies, together with the Internet of Things (IoT), are currently being applied to the fundamental techniques to enhance their performance. The **outermost circle** shows biomedical (blue) and environmental (green) challenges where these technologies are being applied.

Despite the significant progress made in sensors, several challenges remain to be addressed. For instance, there is a need to develop sensors that can detect a wider range of analytes with higher sensitivity and selectivity. Additionally, the integration of these sensors with other devices with easier sample preparation and technologies such as wireless communication enabling the Internet of Things (IoT), automation, and energy harvesting remains a challenge. These technological challenges, compounded by the global challenges faced in the biomedical and environmental fields (as depicted in Figure 1), render this area an intriguing subject for further investigation.

In this review, the recent progress in sensors to address biomedical and environmental challenges in the coming years (as shown in Figure 1) is discussed. Specifically, this review will focus on progress over the last five years in micro- and nanotechnology-enabled sensors. We will briefly describe their principles of operation and applications in these fields. We will also identify the current research gaps and future directions in this rapidly evolving field. The review is organized as follows: First, we will provide an overview of micro- and nanotechnology-enabled sensors. Next, we will discuss the applications of sensors in the biomedical and environmental fields, focusing on specific identified challenges (see Figure 1).

The article selection criteria for this review were based on the following factors: relevance to the topic and direct application to the challenges depicted in Figure 1. Preference was given to articles published between 2018 and 2023 to ensure the inclusion of recent research and developments. Priority was given to articles from reputable journals and conferences that had undergone rigorous peer-review processes. Additionally, articles with robust research methodologies, experimental validation, and reliable data were preferred. The selection process aimed to include articles from diverse authors, institutions, and geographical locations to provide a comprehensive view of the research landscape. The exclusion criteria primarily considered timing and availability.

## 2. Innovations Enabled by Nanotechnology in Sensing Techniques

There are several sensing techniques that can be utilized for signal detection (see Figure 1 innermost circle (gray)). This section will present these mechanisms and highlight recent advancements facilitated by nanotechnology (see Figure 1 middle circle). The selection of a particular mechanism depends on the specific application requirements, such as the nature of the signal being detected, the desired sensitivity and accuracy levels, and the environmental conditions in which the sensor will operate.

### 2.1. Resistive Sensing

Resistive sensing is a method of measuring physical parameters, such as pressure, force, or temperature, by detecting changes in electrical resistance. This technique is based on the principle that the electrical resistance of a material changes when it is subjected to an external stimulus. This technique is simple and low-cost. However, resistive sensing can be affected by factors such as temperature changes and mechanical wear and tear, which can affect the accuracy of the measurements over time.

**Figure 2:**
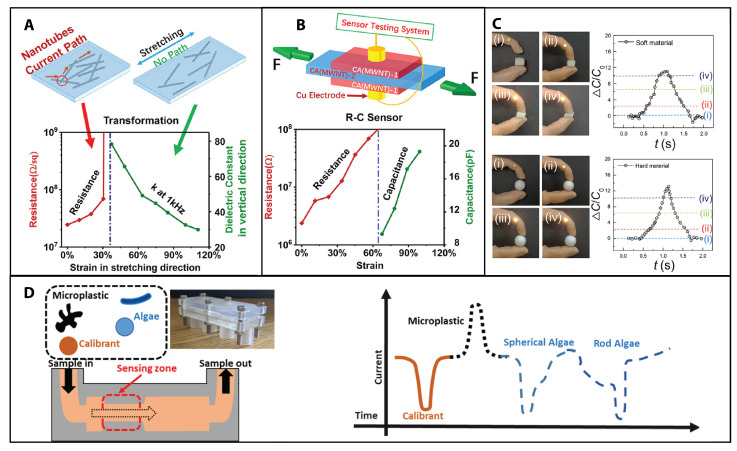
Resistive, capacitive, and resistive pulsing sensors. (**A**) The device can be transformed from conductive to non conductive Electrical and dielectric results of nanocomposites of MWNTs and PDMS with various weight fractions. When W(MWNT) = 8.03%, transformation occurs due to tensile strain. (**B**) Schematic showing the integrated R-C strain sensor and characterization method and the performance of the R-C sensor. Reprinted (adapted) with permission from [52]. Copyright 2020 American Chemical Society. (**C**) Capacitive pressure sensor based on porous Ecoflex-multiwalled carbon nanotube composite (PEMC) structures, with a sensitivity (6.42 and 1.72 kPa $\times \;10^{-1}$ in a range of 0–2 and 2–10 kPa, respectively) due to the synergetic effect of the porous elastomer and the percolation of carbon nanotube fillers. The figure shows a 3D model of a constructed prosthetic arm with an integrated robot finger for grasping movements; an LED is attached to the index finger to gauge the pressure sensed by the PEMC-based pressure sensor embedded into the thumb. Demonstration of the grasping abilities of the robot finger for a soft material (top 4 insets), and a hard material (plastic ball; bottom 4 insets), for both cases i-iv: Demonstrate progressive and reversible increase of capacitance as a function of robot finger force. Reprinted (adapted) with permission from [67]. Copyright 2020 American Chemical Society. (**D**) A low-cost and high-throughput multi-use resistive pulse sensor (RPS) produced through additive manufacturing demonstrated the ability to selectively detect and characterize both microplastics (shed from tea bags) and two algae species. Reprinted (adapted) with permission from [55]. Copyright 2020 American Chemical Society.

Nanotechnology has brought many different advances to the field of resistive sensing through the development of different techniques, such as the resistive measurement of nanostructured materials for gas-sensing applications [51], nanocomposites for resistive strain sensors using multiwalled carbon nanotubes (MWNTs, diameters 8–15 nm) [52] (Figure 2A), or the development of resistive pulse sensors (Coulter counter principle), which have been used to characterize everything from whole cells to small molecules [21,22]. In the work of Feng et al. [52], a method to transform conductive nanocomposites into dielectrics using tensile strain was developed. The main application of this phenomenon is integrated resistive–capacitive strain sensors, which show potential in e-skin applications. Additionally, resistive sensing can be used in aqueous solutions to investigate the fluid composition. There is a recent study on a resistive pulse sensing device with nanochannels embedded using nanotechnology to enable label-free biomolecule and bionanoparticle analysis, showing a promising sensing strategy that is not only capable of label-free analysis for nanoscale biomolecules and bionanoparticles but also cost-effective for large-scale manufacturing [53]. Recently, using solid-state nanopores, the dynamics of knots in double-stranded DNA under unique regimes of nanometer-scale confinement, large forces, and short time scales were studied in order to investigate the static and dynamic properties of biopolymers [54]. In terms of environmental applications, a tunable 3D-printed microfluidic resistive pulse sensor was developed for the characterization of algae and microplastics [55], showing promising results. See Figure 2C. On the other hand, a study on high-performance resistive humidity sensors based on Ag nanoparticles (AgNPs) decorated with graphene quantum dots (GQDs) showed that a 1:1 ratio of the GQD/AgNP nanocomposite exhibited the best humidity response of 98.14%, with an exponential relation in the humidity range of 25–95% relative humidity at room temperature, as well as faster response/recovery times than a commercial one in the same conditions [56].

### 2.2. Capacitive Sensing

Capacitive sensing is a technique that has the ability to detect and gauge the presence or absence of virtually any type of object, irrespective of its material composition. This technology operates as a non-contact method by utilizing the unique properties of an electric capacitor and its electrical field. Capacitive sensors are able to create a highly sensitive system that can detect even the slightest changes in the electric field.

Capacitive pressure sensor arrays have been applied to electronic skin, medical prosthetics, wearable devices, biometrics, touch pads, and touch screens [57]. Recent progress in capacitive sensing includes the development of flexible wearable devices with high sensitivity and wide ranges [58,59]. There has also been progress in pressure sensors for wearable electronics, including porous pyramidal PDMS dielectric layers [60].

Finally, Cao et al. presented a review of conductive polymer composites with different micro/nano-conductive network structures based on the fundamental tunneling percolation theory and their potentialities and drawbacks for tactile sensor applications. They highlighted how model simulations can be used to clearly elucidate the structure-and-property relationship and guide the modulation of the network structure of conductive composites, discussing how emerging machine learning paradigms may create newly conductive polymer composites in the future [61].

### 2.3. Piezoelectricity Sensing

In a piezoelectric material, the material generates an electric potential in response to a deformation. This property can be used for sensing and actuation. Recent advances in nanotechnology for piezoelectric sensing include the development of biosensors for detecting heavy metals [62]. Piezoelectric thin films such as zinc oxide and aluminum nitride have found applications in acoustic biosensors [63].

Piezoelectric-based biosensors utilize bioreceptors attached to materials such as ceramics and quartz. These materials generate a measurable signal in response to mass-induced oscillations on the surface of the piezoelectric crystal, enabling the detection of low concentrations of lead ions [62].

In an emerging application, piezoelectric, photoexcitation, and semiconductor properties can be coupled in two-way or multiway interactions, leading to the emergence of new research fields, such as piezotronics and piezo-phototronics. These areas of research have garnered significant attention due to their potential applications in utilizing basic mechanical stimuli and designing new strain sensors based on the alteration of semiconductor properties caused by strain [64].

On the other hand, Du et al. [65] comprehensively discussed the latest advancements in hydrogel-based piezoelectric devices for biomedical applications. The article particularly emphasizes the potential of such devices in wearable sensing technology, including biosignal sensing, energy harvesting, wound healing, and ultrasonic stimulation. The authors provide a comprehensive overview of the current state-of-the-art research in this field, highlighting the key challenges and opportunities for future development. On a similar topic, a composite hydrogel with piezoresistive and piezoelectric sensing for flexible strain sensors with enhanced sensitivity and a wide frequency band response for smart wearable strain sensors was reported by [66].

These advances in nanotechnology have enabled the development of piezoelectric sensors with improved sensitivity, stability, efficiency, and affordability. The use of nanotechnology has allowed for the creation of piezoelectric sensors that can be used for a wide range of applications, including structural health monitoring, force sensing, and energy harvesting.

### 2.4. Thermoelectricity Sensing

The thermoelectric effect, which converts heat energy into electrical energy, can be achieved through any of the three fundamental phenomena: the Seebeck effect, the Peltier effect, and the Thomson effect. These phenomena are essential for the development of thermoelectric materials and can be used as sensing techniques [68].

The Seebeck effect refers to the phenomenon in which a temperature gradient (ΔT) across a thermoelectric material can produce the direct conversion of heat energy into electrical energy. The Peltier effect is characterized by the absorption or release of heat at the interface of two different materials as a result of the passage of an electric current. The Thomson effect is observed when a uniform conductor is subjected to a constant current, and the application of a temperature gradient causes the absorption or release of additional heat.

Recent progress in thermoelectric sensing includes advances in materials and device designs for wearable technology [69], computational methods for discovering new thermoelectric materials [70], and the use of two-dimensional heterostructures for thermoelectric applications [71]. Flexible thermoelectric devices have also been developed to convert body heat into useful energy [72].

Thermoelectric sensing has a wide range of applications, including biomedical, thermal-cycling, optical, and sensor applications in the telecom, automotive, consumer, and even power generation industries [9]. Thermoelectric generators are also used in sensors that monitor the vital signs, movements, and physiological conditions of the body in a noninvasive way [9]. Thermoelectric generators (TEGs) are attractive candidates for power sources or self-powered heat sensors for portable devices [73].

**Figure 3:**
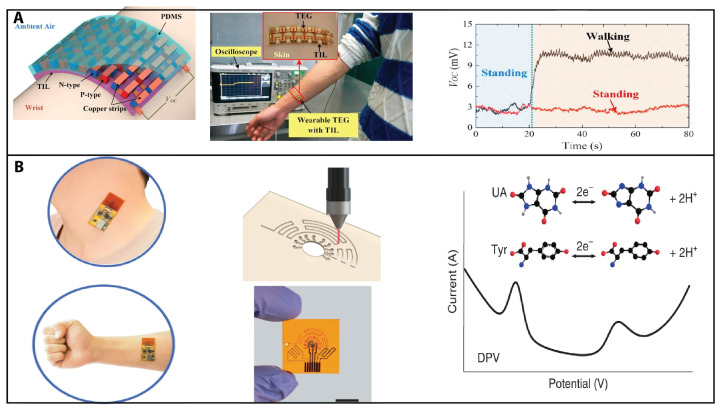
Thermoelectric sensing and electrochemical sensing. (**A**) Wearable thermoelectric generators (TEGs) are attracting interest due to their ability to self-power these electronic devices or sensors by harvesting human body heat. Wang et al. developed a numerical model to investigate the performance of wearable TEGs on the curved human wrist. Reprinted from [74] with permission from Elsevier Copyright (2017). (**B**) Combining microfluidics and laser-engraved fabrication methods, Yang et al. developed a wearable sensor for sensitive detection of uric acid and tyrosine in sweat. Reprinted from [34] with permission from Elsevier Copyright (2020).

Wearable thermoelectric generators (TEGs) are attracting interest due to their ability to self-power these electronic devices or sensors by harvesting human body heat. Wang et al. developed a numerical model to investigate the performance of wearable TEGs on the curved human wrist [74]. See Figure 3A.

The use of nanotechnology has enabled the creation of thermoelectric devices with improved sensitivity, stability, and efficiency, which can be used to measure temperature with improved accuracy and precision.

### 2.5. Acoustic Sensing

Acoustic sensing refers to the use of technology to detect, analyze, and measure sound waves or acoustic signals in various environments and is used to detect a wide range of sounds, from human speech and music to machinery noise and environmental sounds. Acoustic sensing technology typically consists of a sensor that can detect sound waves and can convert them into an electrical signal, which is then processed and analyzed using specialized software or hardware. The sensor may be a microphone or a more complex device, such as an acoustic resonator or a piezoelectric sensor.

Recent advances in nanomaterial-enabled acoustic devices have greatly improved the sensitivity, tunability, and other limitations of conventional acoustic sensors; however, further exploration of flexible acoustic devices is a key priority and will have a great impact on the advancement of intelligent robot–human interaction and flexible electronics [75]. Wei et al. presented a review of nanomechanical resonators, which are able to detect very small changes in acoustic waves [76]. The coupling of micro- or nanomechanical resonators via a shared substrate is intensively exploited to build systems for fundamental studies. This can be exploited to realize high-quality phonon cavities, an alternative to acoustic radiation shielding, acoustic superlenses, waveguides, vibration attenuation, mass sensing, and phononic graphene [77].

Other applications use nanowire-based acoustic sensors, which are a type of sensor that incorporates nanowires. A soft resistive acoustic sensor based on suspended nanowires has also been developed for future wearable voice recognition devices, cochlear implants, and human–machine interfaces [78]. A flexible, localized acoustic sensor with a mass block-beam using silver nanowires coated on polydimethylsiloxane (PDMS) has also been designed to test the sound source orientation [79]. Nanowire-based sensors have been studied in the context of 1D nanostructures, nanowire synthesis for sensing, and composite nanowire materials to determine the freshness status of mackerel fish (*Scomber scombrus*) in a quick and noninvasive way [80]. In terms of fabrication, nanocrystalline diamonds are ideal materials for manufacturing various microelectromechanical devices and mass sensors due to their high-frequency working range and excellent material properties. Recently, a low-cost method to fabricate diamond-based micro-resonators using a modified home/office desktop inkjet printer was used to locally deposit nanodiamond ink as ϕ 50–60 μm spots [81].

### 2.6. Electrochemical Sensing

Electrochemical sensing is a technique that uses electrochemical principles to detect and measure the concentration of a specific chemical species in a sample. Electrochemical sensors typically consist of a three-electrode cell: an interrogating electrode, a reference electrode, and a counter electrode. A signal is generated within the electrochemical cell and then measured and analyzed by a potentiostat [82]. Nanotechnology has brought significant progress to the field of electrochemical sensing by enabling the development of highly sensitive and selective sensors with improved performance characteristics.

One of the main advantages of nanotechnology in electrochemical sensing is the ability to increase the surface area of the electrode, which enhances the sensitivity and selectivity of the sensor. This can be achieved by using nanomaterials such as carbon nanotubes, graphene, and metal nanoparticles to modify the electrode surface [82].

Nanotechnology has also enabled the development of new sensing mechanisms, such as surface-enhanced Raman spectroscopy (SERS) and electrochemiluminescence (ECL), which offer high sensitivity and selectivity for certain analytes [18].

Recent advancements in electrochemical sensors include the development of microfluidic-based electrochemical sensors for sensing foodborne pathogens [18], advances in electrochemical sensing platforms enabling the detection of biomolecules and whole cells [23], and the integration of a flexible electrochemical sensor into a microfluidic chip for simulating and monitoring vascular mechanotransduction [83].

For other applications, various nanomaterials, such as carbon nanotubes, graphene, and metal nanoparticles, have been integrated into electrochemical sensors to enhance their performance, such as sensitivity, selectivity, and stability [24,84,85].

For wearable applications, electrochemical sensors have been developed using microfluidics and nanotechnology to monitor various physiological parameters, such as glucose, lactate, and pH in real time and even lead and cadmium from a sample sweat [86]. These sensors are noninvasive and comfortable to wear, and some can transmit data wirelessly to a smartphone or a cloud server, as discussed by [87,88].

On the other hand, paper-based electrochemical sensors have been developed using microfluidics and nanotechnology for point-of-care diagnosis in resource-limited settings. These sensors are low-cost and portable and can detect various analytes, such as glucose, cholesterol, and DNA. Recent advancement are ePADs (electrochemical paper-based analytical devices), including several preparation methods [31] and printed paper-based electrochemical sensors for low-cost point-of-care disease and environmental diagnostics [32]. The development of electrochemical and optical detection methods based on microfluidic paper-based analytical devices (μPads) for point-of-care testing applications is discussed by Hou et al. [33]. Combining microfluidics and laser-engraved fabrication methods, Yang et al. developed a wearable sensor for the sensitive detection of uric acid and tyrosine in sweat [34]. See Figure 3B.

Lastly, multiplexed electrochemical sensors have been developed using microfluidics to detect multiple analytes simultaneously. These sensors can be used for the high-throughput screening of samples and can reduce the cost and time required for analysis [88]. Multiplexing sensing refers to the simultaneous detection of multiple analytes using a single sensor platform. This approach offers several advantages, such as reduced analysis time, increased throughput, and cost-effectiveness compared to traditional single-analyte detection methods [34]. Multi-analyte electrochemical detection can enhance the efficiency of analyzing multiple food safety hazards. However, interference and cross-reactions among analytes can impede simultaneous detection by electrochemical sensors. This interference can arise from similar analytes, interferences between channels, and low detection sensitivity. To address these challenges, different nanostructuring options can be employed. These include incorporating nanoparticles to enhance electrocatalytic properties, utilizing nanostructured electrodes to improve sensitivity and selectivity, depositing nanostructured films or coatings to enhance selectivity and stability, incorporating nanopores or nanochannels for controlled analyte diffusion, and employing nanostructured catalysts to enhance electrochemical reactions. These nanostructuring approaches mitigate interference and improve the performance of electrochemical sensors in the simultaneous detection of analytes [89,90,91,92]. Some recent advancements in multiplexing sensing include the development of an antifouling coating that enables affinity-based electrochemical biosensing in complex biological fluids [93].

Overall, these advancements in electrochemical sensors hold promise for a wide range of applications in healthcare, environmental monitoring, food safety, and many other areas.

### 2.7. Optical Sensing

Optical sensing refers to the process of detecting and measuring light or other forms of electromagnetic radiation using specialized sensors or devices. Optical sensing works by detecting changes in the intensity, wavelength, polarization, phase, or direction of light or other forms of electromagnetic radiation. Common types of optical sensors include photodiodes, phototransistors, photovoltaic cells, and charge-coupled devices (CCDs).

#### 2.7.1. Photodetection Sensing

A photodetector (PD) is an optoelectronic device that converts incident light or other electromagnetic radiation in the UV, visible, and infrared spectral regions into electrical signals [94]. Nanotechnology has brought recent progress to photodetection sensing by enabling the development of low-dimensional photodetectors [95] and nanostructured photodetector technology for UV sensing and pollution detection [96]. There are also recent developments in lead-free double perovskites for X-ray and UV-vis photodetection [97].

Graphene and semiconductor nanocrystals have been used in the development of photodetectors due to their unique optical and electronic properties, such as high absorption coefficients and high charge carrier mobility. In this context, nanostructured materials have been developed, such as perovskites, which have a long carrier lifetime, high carrier mobility, and facile synthesis [98]. Low-dimensional semiconductor/Si hybrid heterostructures provide a great platform for fabricating high-performance photodetectors [99]. Various absorber materials, such as quantum dots (QDs), plasmonic metal nanoparticles, perovskites, and organic materials, have been investigated to improve broadband absorption [100].

#### 2.7.2. Plasmonic Nanosensors

Microdevices and nanotechnology have had a significant impact on the field of optical sensing by enabling the development of more sensitive and selective sensors with higher resolution and faster response times. The use of nanoscale materials, advanced fabrication methods, and surface plasmon resonance (SPR) in plasmonic nanosensors has enabled the development of highly sensitive and selective sensing technologies for a wide range of applications, including biomedical diagnostics, environmental monitoring, and food safety [101,102].

SPR is a phenomenon that occurs when light interacts with a thin metal film or nanoparticle at a specific angle, causing the conduction electrons in the metal to oscillate and generate an electromagnetic field known as a surface plasmon. This electromagnetic field is highly sensitive to changes in the refractive index of the surrounding environment, making SPR a popular technique for sensing applications [103].

Plasmonic biosensors use evanescent waves to detect analytes such as proteins, nucleic acids, pathogens, and drugs without the need for labeling. This label-free detection provides exceptional sensitivity in a real-time one-step format [104].

Plasmonic nanosensors take advantage of the SPR phenomenon to detect changes in the environment. By functionalizing metal nanoparticles with specific biomolecules or chemical groups, plasmonic nanosensors can detect the presence of specific analytes or molecules in a sample. The interaction between the analyte and the functionalized nanoparticles causes a shift in the SPR signal, which can be measured to determine the concentration or presence of the analyte [25].

Recent advancements in surface plasmon resonance (SPR) include the development of highly sensitive pathogen biosensing techniques [105]. There have been significant developments in SPR sensing devices for the detection of various chemical and biological molecules [26]. A review by Huo et al. describes the principle of SPR imaging and discusses recent developments in prism-coupled and non-prism-coupled SPRI techniques in detail [106].

Knoezer et al. demonstrated how an optical frequency comb can be used to enhance the functionality of an integrated photonic biosensor platform by sampling the spectral response of a Mach–Zehnder interferometer and arranging the sample response to periodic intervals to combine them into a single measurement of the interferometer phase, resulting in a lower limit of detection of 3.7 × 10${}^{-7}$ RIU [107].

**Figure 4:**
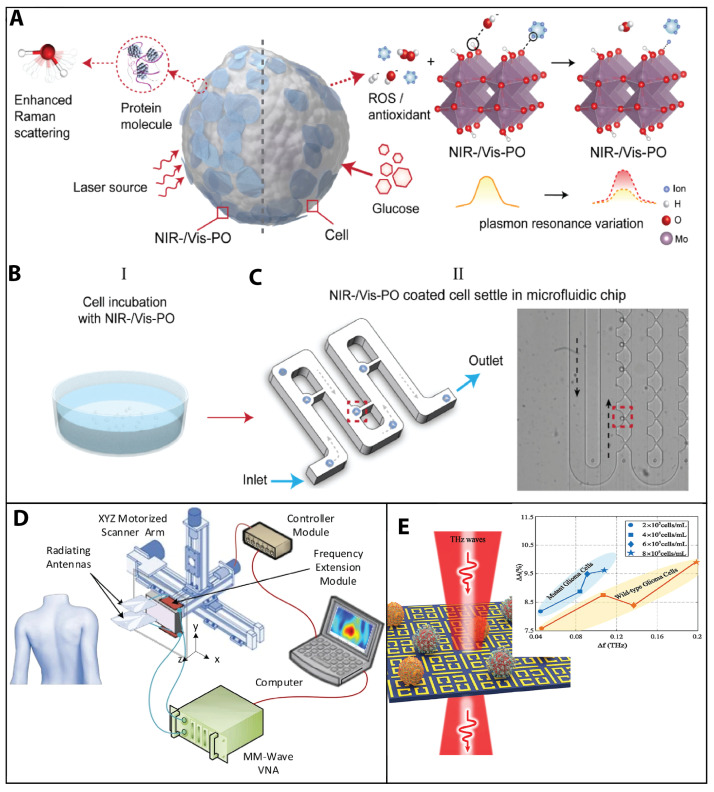
Optical sensing and electromagnetic wave technology. (**A**–**C**) Single-cell analysis using a dual-functional nanoprobe based on dopant-driven plasmonic oxides, which enables the identification of a single THP-1 (related to leukemia) cells from peripheral blood mononuclear cells (PBMCs) and human embryonic kidney cells from human macrophage cells based on their surface-enhanced Raman spectroscopy (SERS) patterns. (**B**,**C**) (I) Cells are coated with PO (plasmonic oxide) via incubation. (II) Illustration (**left**) and optical image (**right**) of the PO-coated cells infused into a microfluidic chip and settled separately in the small grooves (highlighted in red). Reprinted from [108] with permission from Elsevier Copyright (2022). (**D**) Millimeter-wave technology. Schematic of the developed ultra-wideband millimeter-wave imaging system for real-time in vivo skin cancer imaging, achieving an overall synthetic bandwidth of 98 GHz. At each scanning step, two sub-band antennas are placed in front of the target, transmit their signals in their respective sub-band frequency ranges, and record the backscattered signals. Reprinted from [110] under Creative Commons Attribution 4.0 International License Copyright (2022). (**E**) Metamaterial-inspired biosensors using THz detection and a label-free biosensing approach for molecular classification of glioma cells. A metamaterial biosensor consisting of cut wires and split-ring resonators is used to realize polarization-independent electromagnetically induced transparency (EIT) at THz frequencies. The theoretical sensitivity of the biosensor was evaluated up to 496.01 GHz/RIU. The measured results indicated that mutant and wild-type glioma cells can be distinguished directly by observing variations in both the EIT resonance frequency and magnitude at any cell concentration without antibody introduction. Reprinted from [111] with permission from Elsevier Copyright (2022).

In another application, a dual-functional nanoprobe based on dopant-driven plasmonic oxides was recently developed, demonstrating a maximum accuracy above 90% in distinguishing single THP-1 cells (related to leukemia) from peripheral blood mononuclear cells (PBMCs) and human embryonic kidney (HEK) 293 cells from the human macrophage cell line U937 based on their surface-enhanced Raman spectroscopy (SERS) patterns [108]. See Figure 4A.

Lastly, in terms of the tunability of materials, nanoscale materials are commonly used in plasmonic nanosensors to tune the plasmonic properties of the material and increase sensitivity. Researchers can control the size, shape, and composition of nanoparticles to optimize sensor performance, and the high surface-area-to-volume ratio of nanoscale materials allows for their functionalization. Two-dimensional nanomaterials such as graphene and transition metal dichalcogenides have been studied for their potential to improve SPR sensor performance with increased sensitivity and narrower full-width half-maxima [26]. Similarly, metal–organic frameworks provide many attractive features, including tailorable porosity, high surface areas, good chemical/thermal stability, and various host–guest interactions, making them appealing candidates for volatile organic compound capture and sensing [109].

#### 2.7.3. Photonic Crystals

Photonic crystals are periodic structures with a periodicity on the order of the wavelength of light. By controlling the periodicity and composition of photonic crystals, it is possible to create sensors that are highly sensitive to changes in the refractive index of a sample. For example, photonic crystal sensors have been used to detect small changes in the concentration of glucose in blood samples [112]. The sensor is able to perform at an ultrahigh sensitivity of 4200 nm/RIU, is a low-cost, tunable design, enables real-accurate detection, and has a simple structure, which is supportive of an industrial design using low-cost product nanofabrication techniques. On the other hand, Boes et al. presented an excellent review of lithium niobate, showing its capabilities to open a new-generation field in sensor development due to its virtues, such as a large dynamic range [113]. Additionally, integrated photonics on thin-film lithium niobate holds great promise for realizing low-cost and scaled optical solutions for communication, sensing, and computation [113].

Overall, nanotechnology has allowed for the development of optical sensors with higher sensitivity, selectivity, and resolution, making them valuable tools for a wide range of applications, from biomedical diagnostics to environmental monitoring.

### 2.8. Electromagnetic Wave Technology

Millimeter waves, microwaves, and terahertz waves have emerged as as valuable segments of the electromagnetic spectrum for a wide range of sensing applications addressing biomedical and environmental challenges through their unique sensing capabilities.

In the biomedical field, these waves offer noninvasive and precise methods for imaging, diagnostics, and monitoring. Millimeter waves, ranging from 30 to 300 gigahertz (GHz), are employed for the imaging of skin lesions, the early detection of breast cancer, and noninvasive glucose monitoring [10,110]. Microwaves, spanning the frequency range of 300 megahertz (MHz) to 30 GHz, facilitate deep tissue imaging, thermotherapy, and vital sign monitoring. Terahertz waves, with frequencies between 0.1 and 10 terahertz (THz), have the potential to revolutionize biomedical imaging by revealing molecular-level details and enabling the early detection of diseases [114,115]. In this context, Mirbeik et al. [110] evaluated the application of a real-time high-resolution millimeter-wave imaging (HR-MMWI) system for in vivo skin cancer diagnosis, achieving high diagnostic accuracy (97% sensitivity and 98% specificity) comparable to clinical examination and other methods using a combination of 3D principal component analysis and multilayer perception classification. See Figure 4D. Zhang et al. [111] proposed a label-free biosensing approach using metamaterial-inspired biosensors to classify glioma cells based on variations in resonance frequency and magnitude, achieving high sensitivity and the potential for the molecular classification of glioma cells without antibody introduction. See Figure 4E.

On the other hand, in the environmental realm, these waves find applications in remote sensing, pollution monitoring, and climate research, as well as the identification and analysis of specific chemical compounds and environmental pollution detection [116,117]. Frau et al. developed a novel technique using microwave spectroscopy and planar sensors for the real-time in situ monitoring of water quality, specifically targeting trace metals in polluted mining areas, and demonstrated the immediate response and classification of water pollution levels based on multiple resonant peaks in the GHz range, which represents a significant advancement in quantifying pollutants in water [116].

### 2.9. Field-Effect Transistor (FET) Sensing

Field-effect transistor (FET) biosensors are a type of biosensor that use a FET as the transducer to convert the biological signals generated by the interaction of a biological molecule with its ligand into an electrical signal [47].

The basic structure of a FET biosensor consists of gate, source, and drain electrodes and a semiconductor channel between them. The gate electrode is typically modified with a receptor molecule that can selectively interact with the target analyte of interest. When the analyte binds to the receptor on the gate electrode, it induces a change in the electrical charge at the interface between the gate and the semiconductor channel, resulting in a change in the current flowing through the channel [47]. Recent progress in field-effect transistor (FET) biosensors has been made in the early detection of biomarkers for different diseases, including cancer and infectious diseases [118,119]. Sadigh et al. highlighted the advantages of FET-based biosensing arrangements for early biomarker detection and drug screening [120]. The advantages of using FET biosensors in early biomarker detection include the specific and sensitive detection of cancer biomarkers, which can be used to detect early-stage cancer, monitor the progress of the disease, and evaluate the effectiveness of therapies [121]. FET biosensors are attractive pocketable devices with short detection time capabilities [118,119]. They can detect diseases rapidly and reliably [120]. Some of the notable progress made in this field includes:

Nanopores in FET biosensors. Researchers have developed FET biosensors with nanopores, which can enhance the sensitivity and selectivity of the device by increasing the surface area available for biomolecule binding. A new class of nanoscale sensors called nanopore extended field-effect transistor (NEFET) has been designed for selective single-molecule detection, enabling higher molecular throughput, an enhanced signal-to-noise ratio, and heightened selectivity via functionalization with an embedded receptor [122].

Graphene-based FET biosensors. Graphene, a two-dimensional material, has shown promise in improving the sensitivity and selectivity of FET biosensors. Graphene-based FET biosensors have been shown to detect single molecules of DNA and proteins. Recent progress has been made in the design and development of high-performance graphene-based FET biosensors. A review of recent advances in the general design strategy of high-performance graphene field-effect transistor (GFET) biosensors was conducted, with an emphasis on their sensing performance and characteristics [123]. Graphene-based FET biosensors decorated with Pt/Pd nanoparticles have also been successfully utilized for the rapid detection of COVID-19 [124], with a limit of detection (LOD) of 1 fgmL of the COVID-19 spike antigen.

Microfluidic integration. FET biosensors have been integrated with microfluidic systems, enabling the real-time label-free analysis of biological samples with high sensitivity and specificity using fully closed devices [125].

These advancements in FET biosensors hold great potential for their use in a range of applications, from medical diagnostics to environmental monitoring, and could lead to significant improvements in disease detection and treatment, as well as in the development of new drugs and therapies.

### 2.10. Biosignal Sensing

Biosignals (or bioelectrical signals) are any signals in living beings that can be continually measured and monitored, such as a beating heart or a contracting muscle [126]. Biosignals can be emitted by our body when flexing muscles, blinking eyes, or even thinking. They originate due to the physiological processes in living beings [126].

Nanotechnology has enabled significant advances in soft and dry electrodes for wearable electronics for biosignals, such as the electroencephalogram (EEG), electrocardiogram (ECG), and electromyogram (EMG) [127]; thin-film electrodes based on two-dimensional materials [128]; and advances in biosignal sensing and signal processing [129] improving the signal-to-noise ratio and sensitivity. On the other hand, there are also new devices for home sleep monitoring that incorporate miniaturized electronics [130,131].

**Figure 5:**
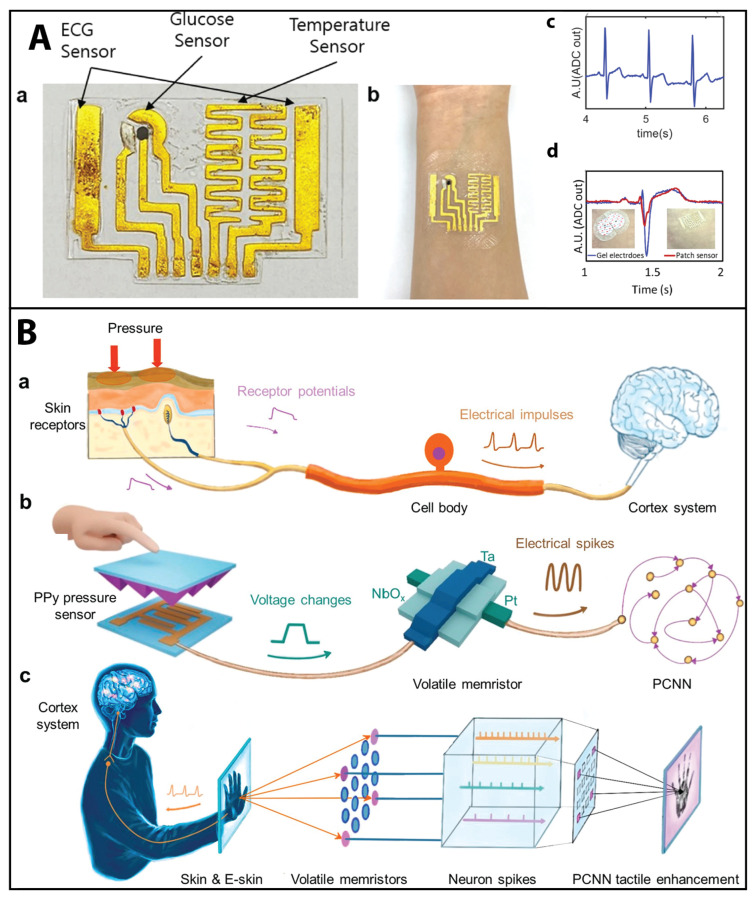
Biosignals (**A**) and bioinspired sensing (**B**). (**Aa**,**Ab**) Flexible and stretchable sensor platforms of biosignals for glucose, electrocardiogram, and body temperature. (**Ac**) Recorded ECG signals using the proposed ECG sensor and magnified view clearly showing PQRST waveforms. (**Ad**) ECG signal measured from the patch sensor attached to the chest of a human subject, compared to the signal from conventional gel-type electrodes. Reprinted from [135] under Creative Commons Attribution 4.0 International License Copyright (2021). (**B**) Bioinspired sensing. Biological and artificial mechanoreceptors. (**Ba**) Biological mechanoreceptors convert specific types of external pressure stimuli into receptor potentials. The soma of the mechanoreceptor integrates potentials and generates electrical impulses. The coded pressure information is ultimately sent to the brain for further processing. (**Bb**) The artificial mechanoreceptor is composed of a micro-pyramidal polypyrrole resistive pressure sensor and a NbOx volatile memristor. The output electrical spikes can be processed effectively by a pulse-coupled neural network (PCNN). (**Bc**) Schematic illustration of the tactile perception process in humans (**left**) and in the artificial mechanoreceptor system enhanced by the PCNN (**right**). Reprinted from [138] with permission from ACS Copyright (2021), American Chemical Society.

Furthermore, the field of wearable devices for the continuous monitoring of biosignals has become increasingly popular [132,133]. These devices can continuously collect high-fidelity biosignals over weeks and months at a time. Advances in nanotechnology have enabled the development of wearable systems capable of monitoring the previously discussed biosignals as well as oxygen saturation and motion (with applications to breathing rate and pulse detection on small arteries) [133]. Recently, wearable sensors based on colloidal nanocrystals (NCs) have been developed for the monitoring of human motion, strain, pressure, and temperature with unprecedented accuracy, reliability, and tunable properties; however, challenges still exist in terms of developing advanced patterning techniques that can take advantage of advanced fabrication methods, such as direct optical lithography using light-responsive ligands without photoresists, for the realization of practical and cost-efficient NC-based wearable devices [134]. Kim et al. [135] reported a microfabrication process for flexible and stretchable sensor platforms of biosignals encompassing conductor formation and patterning to encapsulate and open sensing windows. The feasibility of the method was demonstrated in the sensing of electrochemical (glucose), electrical (electrocardiogram), mechanical (strain), and thermal (body temperature) modalities. See Figure 5A.

Krishnan et al. developed a wireless, battery-free epidermal electronic device for the continuous thermal characterization of the skin. They demonstrated its capabilities in measuring skin hydration and injury, and importantly, the technology is capable of data acquisition without disrupting natural daily activities [136].

### 2.11. Bioinspired Sensing

In recent years, the field of bioinspired sensing has observed a multitude of significant advancements. These sensors are rooted in the notion that nature embodies a profound reservoir of inspiration, offering invaluable insights for the development of efficacious solutions to address various sensing challenges.

Li et al. [137] developed a novel biosensor using two-photon polymerization and graphene to fabricate a bioinspired cage structure that enhances the detection of motile bacteria by concentrating them around the sensing region, resulting in significantly enhanced sensing signals for fluorescence, pH, and electrical measurements.

Among different applications of skin-inspired devices, there has been significant progress in artificial mechanoreceptors for tactile enhancement and integration. In this regard, Li et al. [138] developed an artificial mechanoreceptor by integrating a polypyrrole-based resistive pressure sensor with a volatile NbO${}_{x}$ memristor, enabling tactile sensory coding and perception similar to natural skin. The system converts mechanical stimuli into electrical spikes, processes spike frequency characteristics with a pulse-coupled neural network for sensation enhancement, and integrates signals from parallel sensor channels resembling the coding of intensity in tactile neural processing, offering potential for future bioinspired electronic systems. See Figure 5B.

In a similar application of tactile and bioinspired skin, there has been recent progress in developing methods for self-healing electronic skins for aquatic environments [139].

Xu et al. [140] designed a self-powered triboelectric palm-like tactile sensor (TPTS) inspired by the leathery, granular texture of the palms of sea otters, enabling underwater vehicles to build a tactile perceptual system. The TPTS can detect and distinguish normal and shear external loads in real time, approximate the external stimulation area, and maintain stable performance under high-frequency contact, making it a promising tool for integration into grippers mounted on underwater vehicles to perform various underwater tasks. Ji et al. [141] developed a novel hybrid dielectric inspired by human skin, comprising a low-permittivity micro-cilia array, a high-permittivity rough surface, and a micro-dome array, enabling capacitive tactile sensors to achieve an ultrawide linearity range of up to 1000 kPa and a high sensitivity of 0.314 kPa${}^{-1}$ by enabling pressure-induced series-parallel conversion and controllable capacitance, with potential applications in healthcare monitoring and control command conversion. In other applications, bioinspired flow sensors have been the subject of recurrent investigation, and lately, novel methods to create flow sensors have been created, including a bioinspired airflow sensor with self-bended 3D hair-like configurations [142]. Similarly, a bioinspired flow sensing cupula using capacitive sensing with little hysteresis to accurately determine flow rates was recently developed by Wissman et al. [143].

Among various methods using light, bioinspired photonic crystal patterns, such as the identified static patterns of butterfly wings, have been developed for sensing and information display [144,145,146]. For applications for monitoring physiological variables, a disposable bioinspired kirigami fish-based wearable biosensor was recently developed using microfluidic channels and stretchable materials for sweat collection, diagnostics, and motion monitoring [147].

Lastly, in the domain of pressure sensors, Zhao successfully engineered biomimetically textured porous materials, leading to the creation of exceedingly sensitive and flexible piezoresistive structures [148]. These structures exhibit remarkable capabilities in detecting pressure within the range of 140 Pa to 0.5 Pa, thereby enabling the real-time monitoring of various human physiological signals, such as finger pressing, voice vibration, swallowing activity, and wrist pulse [148].

## 3. Overview of Recent Innovations Addressing Different Challenges

In the preceding sections of this article, a comprehensive range of sensing techniques was presented and classified by the fundamental principles of sensing. However, in this section, a distinct approach will be employed to shed light on the current pivotal challenges encountered in the biomedical and environmental domains, as illustrated in the outermost circle in Figure 1. By shifting our perspective from principles to the forefront of research, various noteworthy studies will be presented and discussed.

### 3.1. Biomedical Challenges

**Figure 6:**
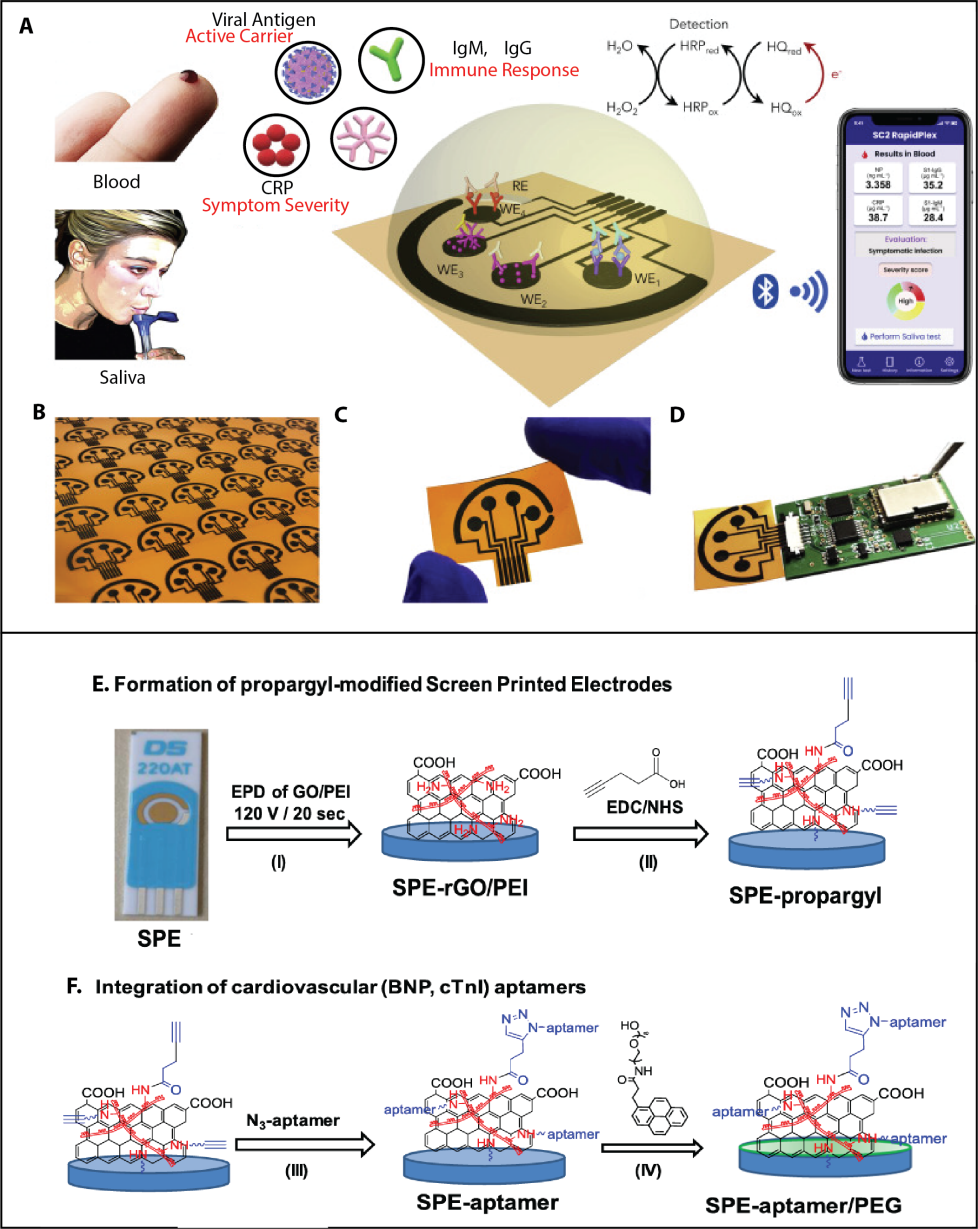
Biomedical challenges. Infectious diseases (**A**–**D**) and NCDs (**E**,**F**). (**A**–**D**) Illustration of the RapidPlex multisensor telemedicine platform. (**A**) Schematic illustration of the SARS-CoV-2 RapidPlex multisensor telemedicine platform for detection of SARS-CoV-2 viral proteins, antibodies (IgG and IgM), and inflammatory biomarker C-reactive protein (CRP). Data can be wirelessly transmitted to a mobile user interface. WE, working electrode; CE, counter electrode; RE, reference electrode. (**B**) Mass-producible laser-engraved graphene sensor arrays. (**C**) Photograph of a disposable and flexible graphene array. (**D**) Image of a SARS-CoV-2 RapidPlex system with a graphene sensor array connected to a printed circuit board for signal processing and wireless communication. Reprinted from [150] with permission from Elsevier Copyright (2020). NCDs—(**E**,**F**) Nanosensors able to detect biomarkers in blood, urine, or saliva that indicate the presence of a cardiovascular diseases, such as troponin, D-dimers, brain natriuretic peptide (BNP), and cardiac troponin I (cTnI). Reprinted from [159] with permission from ACS Copyright (2021), American Chemical Society.

**Figure 7:**
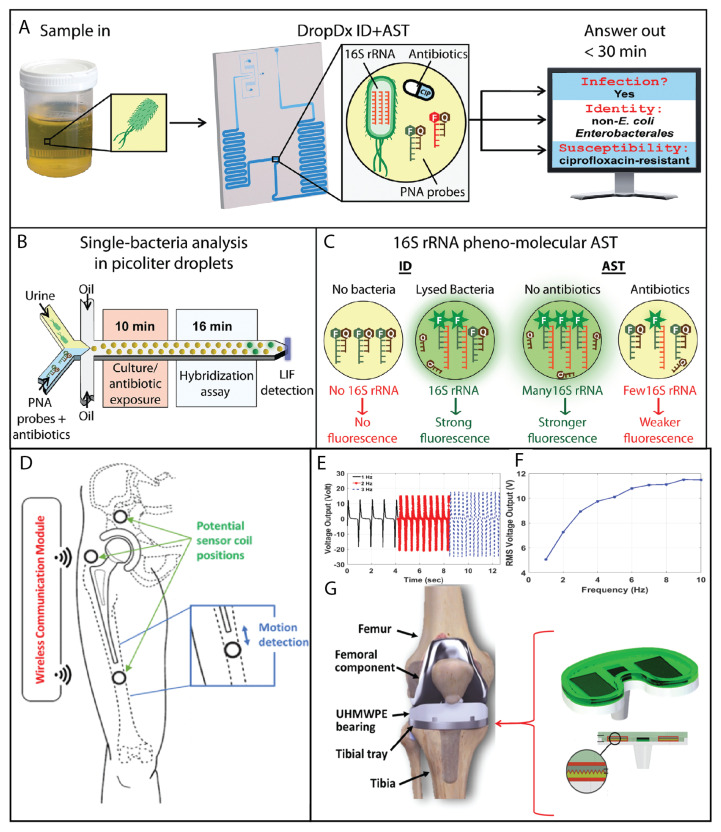
Biomedical challenges. Antibiotic resistance (**A**–**C**) and aging population (**D**–**G**). For antibiotic resistance (**A**–**C**), microfluidics has been successfully applied. Pathogen identification and antimicrobial susceptibility testing (AST) from urine samples were achieved within 30 min, allowing the detection of 16S rRNA from single bacterial cells encapsulated in picoliter droplets and enabling the molecular identification of uropathogenic bacteria directly from urine in as little as 16 min. Moreover, in-droplet single-bacterial measurements of 16S rRNA provide a surrogate for AST, shortening the exposure time to 10 min for gentamicin and ciprofloxacin. Reprinted (adapted) from [36] under the terms of the Creative Commons CC BY license. (**D**–**G**) The aging population will push the development of smart implants. A new wireless inductive proximity sensor system enabled the early detection of implant loosening with high precision. Reprinted (adapted) with permission from [178]. (**E**–**G**) Development of a smart knee implant utilizing triboelectric energy harvesters for self-powering load sensors. Reprinted (adapted) with permission from [179].

Biomedical research and innovation are constantly advancing, and microdevices and nanotechnology are playing an increasingly important role in addressing some of the most pressing challenges in healthcare. The outermost circle (blue) in Figure 1 presents some of the most important challenges identified, including infectious diseases, noncommunicable diseases (cancer, cardiovascular, diabetes, mental health), antibiotic resistance, and the aging population, which can be targeted using frontier technologies offering promising solutions to complex issues. Figure 6 and Figure 7 present some of the most recent progress made in addressing these challenges using micro- and nanotechnologies. Furthermore, the following sections discuss the progress made in tackling the most pressing challenges through the utilization of microdevices and nanotechnology from a sensor perspective.

#### 3.1.1. Infectious Diseases

The emergence of new infectious diseases is a constant threat. In the last 20 years, we have seen the emergence of several new viruses, including SARS-CoV, MERS-CoV, and COVID-19. It is likely that new diseases will continue to emerge in the coming years [149].

Micro- and nanotechnologies are being developed to address infectious disease problems, including the development of new diagnostic tools, therapeutics, and prevention strategies. Here are some examples of current progress:

Rapid diagnostics. Micro- and nanotechnologies are being used to develop rapid diagnostic tests for infectious diseases. For example, researchers are developing nanosensors that can detect viral or bacterial particles in clinical samples, allowing for rapid diagnosis and treatment, particularly in the context of COVID-19 [1,11,12]. This includes the development of new diagnostic tests that are simple, rapid, and sensitive. There have also been advances in using nanotechnology for the detection of other diseases, such as arbovirus-borne diseases [13]. Figure 6A presents a device engineered by Torrente et al. [150], who developed a multiplexed, portable, wireless electrochemical platform called the SARS-CoV-2 RapidPlex by utilizing mass-producible laser-engraved graphene electrodes. This platform enables the ultrasensitive and rapid detection of COVID-19 by detecting the viral antigen nucleocapsid protein, IgM and IgG antibodies, and the inflammatory biomarker C-reactive protein in physiologically relevant ranges, showing promise for high-frequency at-home testing and the telemedicine diagnosis and monitoring of COVID-19.

Tessaro et al. [151] investigated the use of gold spherical nanoparticles (AuNPs) functionalized with oligonucleotides as plasmonic nanobiosensors for the rapid and sensitive detection of SARS-CoV-2 in ready-to-eat vegetables, demonstrating their potential as a low-cost and efficient method using loop-mediated isothermal amplification (LAMP) and ultraviolet–visible (UV-Vis) absorption spectroscopy.

Targeted drug delivery. Sensing technologies, combined with advanced drug delivery systems such as nanoparticles, liposomes, and hydrogels, enable targeted drug delivery with enhanced precision, reduced side effects, and improved therapeutic outcomes of infectious diseases. Yan Li et al. [152] successfully prepared nano-polydopamine-reinforced hemicellulose-based hydrogels with multistage pore structures, exhibiting excellent mechanical properties, stable electrochemical properties, and self-adhesive properties. The hydrogels demonstrated high ultimate tensile strain, strong shear adhesion to skin tissue, improved electrical conductivity, and, remarkably, the ability to detect body movements in real time as a motion sensor. Additionally, they facilitated the loading of cationic drugs and the transdermal introduction of electrically stimulated drug ions, making them potential candidates for next-generation flexible materials suitable for health monitoring and self-administration. On the other hand, liposomes can be modified to respond to specific enzymes present in the target site or associated with certain diseases. Naoto Asai et al. [153] developed an integrated sensor combining quartz crystal microbalance dissipation (QCM-D) and localized surface plasmon resonance (LSPR) to monitor the adsorption and rupture of liposomes in real time. The sensor successfully detected liposome conformational changes upon exposure to Triton X-100 surfactant, providing valuable insights into biomolecular interactions and offering potential applications for monitoring conformational changes in various biomolecules, viruses, bacteria, vesicles, and cells.

Vaccines. Micro- and nanotechnologies are being used to monitor and develop new vaccine technologies for the prevention of infectious diseases. Gepnet et al. [154] conducted a prospective observational study using FDA-approved chest-patch sensors and a mobile application to continuously monitor 13 cardiovascular and hemodynamic vitals post-vaccination with the BNT162b2 COVID-19 vaccine. The study revealed significant and continuous changes in vitals, even in asymptomatic participants, providing evidence of the potential of wearable sensors to revolutionize clinical trials by enabling the earlier detection of abnormal reactions with fewer subjects.

Funari et al. [155] developed a multiplex nanoplasmonic biosensor utilizing gold nanostructures and localized surface plasmon resonance (LSPR) to capture the humoral response in serums against multiple antigens, demonstrating its potential as a cost-effective and high-throughput alternative for antibody profiling in the context of SARS-CoV-2 variants and vaccine development, with successful validation against monoclonal antibodies and murine sera.

Antimicrobial agents. Micro- and nanotechnologies are being used to develop new antimicrobial agents that can be used to treat infectious diseases. Microfluidic platforms have the potential for conducting pharmacological testing of antibiotic susceptibility and toxicity [156]. Recent advances have also been reported in microfluidic-integrated biosensors for pathogen evaluation [157].

Ramki et al. [158] synthesized molybdenum carbide (Mo2C) hexagons and integrated them with functionalized carbon nanofiber (f-CNF) to form a composite. The Mo2C/f-CNF composite demonstrated excellent electrochemical properties and was used to develop an efficient electrode for the ultra-sensitive detection of antimicrobial agents such as metronidazole (MTZ), achieving a low detection limit of 0.002 μM and a linear range of 0.04–647.7 μM. The composite electrode was successfully applied for real-time detection in human urine samples, highlighting its effectiveness for MTZ detection.

#### 3.1.2. Noncommunicable Diseases (NCDs)

Globally, the prevalence of noncommunicable diseases (NCDs), such as diabetes, cancer, cardiovascular disease, and mental health issues, is increasing, which can be attributed to lifestyle changes characterized by unhealthy eating habits, sedentary behavior, tobacco use, and social isolation. Microdevices and nanotechnology have the potential to revolutionize the way that NCDs are diagnosed and treated. Below are different ways in which microdevices and nanotechnology are addressing these challenges.

Early detection. Microdevices and nanotechnology can enable the early detection of NCDs, which can improve patient outcomes. Grabowska et al. [159] developed aptamer-based electrochemical sensors for brain natriuretic peptide (BNP-32) and cardiac troponin I (cTnI), enabling the rapid and accurate detection of cardiovascular abnormalities. See Figure 6E,F. They modified gold-based electrodes with polyethyleneimine/reduced graphene oxide films and immobilized aptamers using Cu(I)-based “click” chemistry. The sensors demonstrated low biofouling, high specificity, and linear responses within clinically relevant concentration ranges, making them promising for point-of-care diagnostics and the early-stage identification of heart failure.

In their review, Pal et al. [2] discuss the importance of the early diagnosis and prevention of cardiovascular disease (CVD) and highlight the limitations of current detection methods for CVD biomarkers. They emphasize the need for a highly efficient, rapid, and sensitive detection method and propose the use of electrochemical biosensors as a promising approach for early detection. The authors also advocate for a multiplex diagnosis approach that incorporates a panel of biomarkers into a single platform, aiming to improve the accuracy and efficiency of CVD diagnosis and facilitate point-of-care applications.

In their opinion review, Abensur et al. [160] presented the results of a survey conducted among physicians to identify relevant biomarkers for emergency situations. They focused on cardiovascular disease and highlighted troponin, D-dimers, and brain natriuretic peptide (BNP) as important biomarkers. They discussed the potential of biosensors, combining medicine, basic research, and engineering, to provide a rapid and reliable analysis of cardiovascular biomarkers, emphasizing the need for multi-marker approaches and machine learning analysis for patient risk assessment. The authors also addressed market access issues related to these biosensor technologies.

Wearable devices. Microdevices can be used to create wearable devices that can monitor patients’ vital signs, such as heart rate, blood pressure, and glucose levels. This can help patients manage their NCDs and prevent complications. Kristoffersson et al. [3] conducted a systematic review of the use of wearable sensors for remote health monitoring and the prevention of noncommunicable diseases (NCDs). They highlighted the potential of wearable sensor systems in detecting warning signals of NCDs, such as stroke and cardiac arrest, and discussed the need for further testing on representative populations to address existing issues and unlock the full potential of wearable sensors in diagnosing, monitoring, and preventing NCDs in the future.

Hatamie et al. [161] conducted a comprehensive review of the emergence, fabrication, materials, and applications of chemical and physical flexible and stretchable textile-based sensors, highlighting their potential in various fields, such as sports performance monitoring, clinical diagnostics, and healthcare applications, and emphasizing the importance of composites and nanomaterials for improved performance and intimate contact with the skin.

#### 3.1.3. Mental Health

Mental health is becoming an increasingly important issue, with more people reporting anxiety and depression [162]. This may be due in part to the stresses of modern life, including work pressures and social isolation. Seunggyu Lee et al. [163] conducted a literature survey on wearable devices and sensors for assessing and monitoring depression. They reviewed 18 studies and examined the types of sensors used (actigraphy units, wristbands, fitness trackers, smartwatches) and parameters measured, discussed future trends and challenges in utilizing wearable devices for the real-time objective monitoring, diagnosis, and treatment of depression, and emphasized the need to address issues such as limited data types, reliability, user adherence, and privacy concerns. Gomes et al. [164] conducted a systematic review of the literature to explore monitoring solutions in mental health using wearable sensors; they discussed the advantages of convenience, cost-effectiveness, and real-world data collection, focused on anxiety, stress levels, and panic attacks, described available sensors and their success in providing meaningful data for machine learning algorithms, and suggested that mental health monitoring through wearable sensors is feasible. Mahsa Sheikh et al. [165] conducted a comprehensive review of the utilization of various sensors embedded in daily life contexts for monitoring mental health. The review explored parameters such as movement, sleep duration, heart rate, electrocardiogram, skin temperature, etc., and their association with psychiatric disorders. The authors emphasized the development of devices with multiple sensors that capture physiological and behavioral data and convert them into features for defining behavioral markers through machine learning. Additionally, the review highlighted the importance of establishing well-defined relationships and considering intrapersonal and interpersonal differences when interpreting the data, ultimately aiming to provide a useful tool for monitoring and managing mental disorders.

#### 3.1.4. Antibiotic Resistance

Antibiotic resistance is a growing problem that threatens to make many common infections untreatable [166]. This is due in part to the overuse of antibiotics in humans and animals. The number of new antibiotics introduced to the clinic is decreasing over time, while the number of resistant strains is increasing [166]. The review by Madhu et al. [167] focuses on recent developments in electrochemical sensing techniques for assessing antibiotic resistance in pathogenic bacteria, highlighting the importance of biorecognition probes, tailor-made nanomaterials, and the potential use of nanomaterials as both transducer electrodes and antimicrobial agents. It also discusses the challenges and future prospects of point-of-care (POC) electrochemical sensors in the healthcare sector. Chunlei Li et al. [168] presented a method for evaluating drug resistance in *Escherichia coli* by using an electrochemical-based sensor that measures the activity of *E. coli* on the electrode surface. They utilized a graphene dispersion to enhance the current signal and assessed the antibiotic resistance of different *E. coli* strains based on the difference in the electrochemical reduction signal when antibiotics are present. Jain et al. [169] developed a novel method for antibiotic susceptibility testing (AST) using a microwave resonator that enables the rapid, contactless, and noninvasive monitoring of bacterial response to antibiotics, demonstrating decisive results in under 6 h and showing the potential for automating the AST workflow in clinical settings. Reynoso et al. [170] presented an overview of novel biosensing strategies for the rapid determination of antimicrobial resistance, highlighting the potential of chemical sensors and biosensors as easy-to-operate, robust, sensitive, specific, and inexpensive tools for both the phenotypic and genotypic detection of resistant microorganisms, with applications in modern healthcare and environmental surveillance. Wang et al. [171] developed culture-free and self-driving DNA nanosensors by combining diffusometry and oligonucleotide probes capable of rapidly and accurately detecting methicillin-resistant *Staphylococcus aureus* by recognizing its genomic DNA sequences, achieving high selectivity and specificity through the use of conjugated fluorescent nanobeads and gold nanoparticles (AuNPs) with a detection limit of 10 pM and offering a promising approach for the rapid and robust detection of superbugs and unknown pathogenic microorganisms. Domingo-Roca et al. [172] developed a fully 3D-printed electrochemical biosensor for rapid bacterial growth monitoring using gel modification and electrochemical impedance spectroscopy. The biosensor allowed for the identification of the growth profiles and antibiotic susceptibility of *Escherichia coli* and *Pseudomonas aeruginosa* within 90 min, providing a faster alternative to the current gold standard of culture-based antimicrobial susceptibility testing, with potential for optimization. The biosensor was demonstrated to be a rapid and cost-efficient platform for phenotypic antibiotic susceptibility testing in clinically relevant concentration ranges, addressing the need for enhanced antibiotic stewardship. Gowers et al. [173] developed a potentiometric microneedle-based biosensor coated with a pH-sensitive iridium oxide layer to detect the levels of β-lactam antibiotics in vivo, demonstrating the potential of this minimally invasive biosensor to provide real-time measurements of antibiotic concentrations, enabling individualized drug dosing, and contributing to a closed-loop therapeutic drug monitoring system.

#### 3.1.5. Aging Population

The aging population poses challenges to biomedical technology, including increased chronic diseases and healthcare needs, financial constraints, and ethical considerations [174]. Smart implants are a promising area of biomedical technology that could help manage chronic conditions while maintaining the independence of the patients [175,176,177]. Smart implants can provide real-time data on health parameters and personalized treatment options, improving the management of chronic conditions and enabling targeted treatments. Examples of smart implants include those designed to monitor heart rate, blood pressure, and other vital signs. These devices can be particularly useful for individuals with complex medical needs, such as those with heart disease or other chronic conditions. These devices can provide real-time data on blood glucose levels, which can be transmitted to a smartphone app or other device to help individuals manage their condition [176]. However, ethical and practical considerations need to be addressed, and not all individuals may be candidates for smart implants. Other examples include cardiac pacemakers, cochlear implants (devices are implanted in the ear to provide hearing to people with severe hearing loss), insulin pumps (devices are implanted in the abdomen to regulate blood sugar levels in people with diabetes), and neural implants (devices are implanted in the brain or spinal cord to treat conditions such as Parkinson’s disease, epilepsy, and chronic pain) [176,177].

To face the problem of detecting prosthetic implant loosening, Mohammad et al. [178] proposed a new wireless inductive proximity sensor system for detecting early implant loosening. The system showed that the designed proximity sensor is capable of measuring the loosening of a hip implant at 50 μm resolution at distances of less than 8 mm and 100 μm resolution at a distance of 15 mm. See Figure 7D.

A smart knee implant using triboelectric energy harvesters was developed by Ibrahim [179], as presented in Figure 7E–G. The system was characterized as a triboelectric energy harvester under compressive body loads. The harvester prototype is intended to be placed between the tibial component and polyethylene bearing of a total knee replacement implant. The results of this work demonstrate that triboelectric energy harvesting is a promising technique for self-powering load sensors inside knee implants. In general, smart implants are likely to be used more frequently in older adults, as the prevalence of many chronic medical conditions tends to increase with age. However, as medical technology advances and smart implants become more widely available, they may be used by a broader range of patients, including younger people with chronic medical conditions.

It is also worth noting that not all smart implants are designed to treat age-related medical conditions. For example, some neural implants are used to treat conditions such as chronic pain or movement disorders, which can affect people of all ages. Similarly, some smart implants are designed to monitor and track health data, rather than treat specific medical conditions, and may be used by people of all ages to improve their health and wellness.

### 3.2. Environmental Challenges

Microdevices and nanotechnology are opening up exciting new avenues for environmental research and innovation. The outermost circle (blue) in Figure 1 presents some of the most difficult challenges in the coming years, including plastic pollution, eutrophication, deforestation, air pollution, climate change, and fatberg management and prevention.

#### 3.2.1. Plastic Pollution

Plastic pollution refers to the accumulation and presence of plastic materials in the environment, particularly in oceans, rivers, lakes, and other natural habitats. It is caused by the improper disposal of plastic waste, the lack of effective recycling programs, and the use of non-biodegradable plastic products. Microdevices and nanotechnology can help address plastic pollution in several ways.

Schmidt et al. [180] conducted a study exploring the potential of multispectral and hyperspectral remote sensing for the detection and differentiation of plastic waste in the environment, using reflectance spectra of commonly used plastic types. They found that while most multispectral sensors were unable to differentiate between plastic types, hyperspectral sensors yielded outstanding results by leveraging narrow shortwave infrared channels with specific absorption bands, highlighting their high capability for plastic differentiation.

Gongi et al. [181] developed an electrochemical-impedance-spectroscopy-based sensor by depositing cyanobacterial extracellular polymeric substances (EPSs) on a gold electrode, enabling the detection of microplastics with a size range of 0.1 µm to 1 mm. The EPS-coated sensor exhibited a highly homogeneous structure with functional groups, allowing for the detection of microplastics at low concentrations, with a low limit of detection of 10–11 M, presenting a new quantification method for microplastics.

Rizzato et al. [182] optimized surface acoustic wave (SAW) delay lines on lithium niobate for environmental monitoring, achieving improved device performance through the optimization of design parameters. They demonstrated the detection of polystyrene particles as nanoparticulates/nanoplastics with a limit of detection (LOD) of 0.3 ng, surpassing the current state of the art.

Secme et al. [183] developed and compared two types of planar microwave sensors, a coplanar waveguide (CPW) and a split-ring resonator (SRR), integrated with optical microscopy to differentiate microscale objects based on their dielectric properties. The standalone microwave sensors were capable of the real-time tracking of relative changes in cellular size and exhibited high signal-to-noise ratios, demonstrating the potential of microwave sensing as a complementary technique for single-cell biophysical experiments and microscale pollutant screening.

Lastly, Sa et al. [184] demonstrated the enhanced sensitivity of flexible screen-printed carbon electrodes (SPEs) for the simultaneous detection of bisphenol-A (BPA), hydroquinone, and catechol in water using a simple electrochemical procedure in acidic solutions, enabling the detection of emerging pollutants from plastic into water to monitoring water quality.

#### 3.2.2. Eutrophication

**Figure 8:**
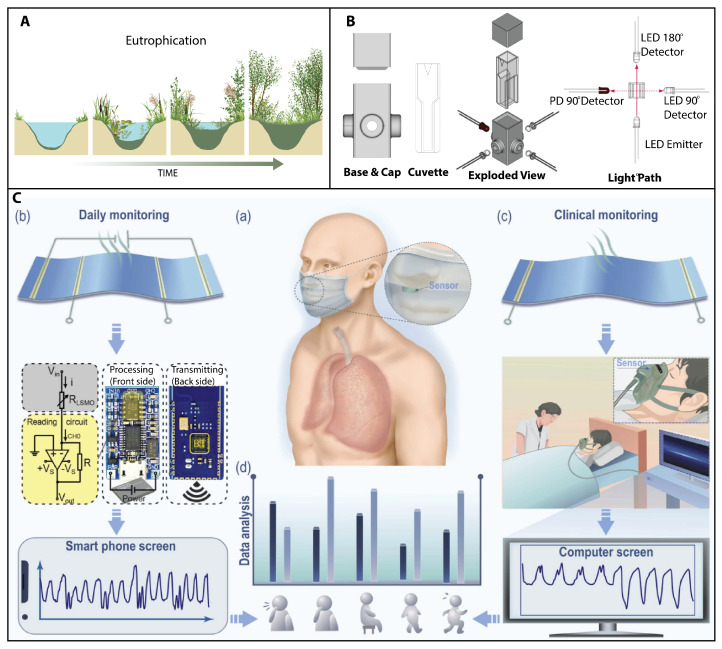
Environmental challenges. (**A**) Eutrophication: Excess nutrients from human activities accumulate in water, causing disproportionate growth of algae and aquatic plants. (**B**) A low-cost and low-power turbidity sensing technique using Paired Emitter–Detector Diodes (PEDDs) adhering to the ISO 7027 turbidity sensing standard, enabling cost-effective and efficient environmental deployments of IoT sensors. Adapted from [186] under an open-access Creative Commons CC BY 4.0 license. (**C**) A flexible respiration sensor integrated into a face mask. (**Ca**) Schematic diagram of monitoring human breath by utilizing manganite oxide respiration sensor. (**Cb**) The wireless mode and (**Cc**) the wired mode of LSMO/Mica sensors used in human breath monitoring. (**Cd**) The obtained data are used for further processing and analysis. Reprinted from [190] under the terms of the Creative Commons CC-BY license.

Eutrophication is a process in which excess nutrients, such as nitrogen and phosphorus, from human activities, such as agriculture and wastewater discharge, accumulate in bodies of water and lead to the disproportionate growth of algae and other aquatic plants [185]. This process can lead to harmful algal blooms, oxygen depletion, and the loss of aquatic life. Sensors in water treatment monitor pH, conductivity, turbidity, dissolved oxygen, disinfectant levels (such as chlorine, ozone, or UV radiation), flow rates, and pressures. They ensure water quality, aid in process control, and optimize treatment efficiency. Figure 8A presents a schematic of eutrophication. Fay et al. [186] developed a low-cost and low-power turbidity sensing technique using Paired Emitter–Detector Diodes (PEDDs), which demonstrated superior performance compared to conventional photodiode–LED arrangements in terms of spectral sensitivity, cost, power use, sensitivity, limit of detection, and adherence to the ISO 7027 turbidity sensing standard, enabling cost-effective and efficient environmental deployments of IoT sensors. See Figure 8B. In a similar application, Rocher et al. [187] developed a low-cost sensor system using infrared and RGB LED light sources and photoreceptors to monitor eutrophication, turbidity, and solid concentration in water bodies, achieving accurate measurements and classification with errors of 7.45% and 11.40%, respectively, and a precision of 89.3% for determining the percentage of algae and an error of 17.95% for determining the levels (mg/L) of algae in water. Recently, a biosensor developed by Marzhocchi et al. [14] based on the reduction of sulfate to H2S by sulfate-reducing bacteria detected H2S formed in freshwater sediment. Enzymatic phosphate ion (Pi) biosensors have also been developed for eutrophication and microbial detection [50].

Lastly, microbial fuel cells (MFCs) are bioelectrochemical devices that generate electricity by using electrons obtained from the anaerobic oxidation of substrates found in wastewater while also removing nutrients [188]. In this regard, Olias et al. [189] developed a self-powered ceramic soil microbial fuel cell (MFC) sensor that monitors dissolved oxygen in water to detect algal growth and potential eutrophication events, showing a correlation between the sensor signal and dissolved oxygen and the algal concentration and highlighting the influence of temperature, dissolved oxygen, nitrates, and pH on sensor performance, making it the first proposed MFC-based biosensor for the in-field, early detection of eutrophic events.

#### 3.2.3. Climate Change

Climate change refers to the long-term changes in the Earth’s climate patterns that have been observed over the past century and are projected to continue into the future [191]. The Earth’s climate has always varied naturally over time, but the current changes are occurring at an unprecedented rate, largely as a result of human activities, such as the burning of fossil fuels, deforestation, and other industrial processes that release large amounts of greenhouse gases (such as carbon dioxide) into the atmosphere [191]. Using micro- and nanotechnology sensors for climate change is crucial due to their distinct advantages, including enhanced efficiency, selectivity, scalability, and versatility, which outperform traditional approaches. Recent advancements in these areas demonstrate the potential of these technologies in tackling climate change challenges.

Solar cells. Solar cells play a vital role in the fight against climate change, as they serve as a renewable power source that reduces greenhouse gas emissions, safeguarding humans, wildlife, and ecosystems and contributing to the mitigation of climate change impacts. The following types of sensors have been recently used in the development of solar cells. Beinert et al. [192] proposed silicon solar-cell-integrated stress and temperature sensors for the direct and continuous in situ measurement of mechanical stress and temperature in photovoltaic (PV) modules, achieving sensitivities of (−47.41±0.14)%/GPa for the stress sensor and (3.557±0.008) $\times 10^{-3}\;K^{-1}$ for the temperature sensor, enabling increased measurement accuracy, process evaluation, reliability tests, and correlation with real exposure to climate in PV module research. Nagasaki et al. [193] developed an optical pH sensor using a pH-sensitive fluorescent dye embedded in a fluororesin membrane filter, enabling the non-destructive measurement of acetic acid and its distribution in a photovoltaic module during damp heat (DH) testing. It successfully tracked pH changes for up to 2000 h and demonstrated a selective response to acetic acid without being influenced by moisture and heat.

Energy storage. Measuring the temperature of supercapacitors and energy storage devices is crucial for ensuring safe operation, optimizing performance, and preventing thermal degradation. For example, Listewnik et al. [194] presented a study in which they demonstrated the integration of a fiber-optic microstructure with a thin zinc oxide coating as a temperature sensor for the in situ monitoring of supercapacitors, achieving a high sensitivity of 109.6 nW $\deg C^{-1}$ and a correlation coefficient of $R^{2}=0.97$ in the temperature range of 30 °C to 90 °C.

#### 3.2.4. Air Pollution

Air pollution refers to the presence of harmful substances in the air we breathe, which can have negative impacts on human health, the environment, and the climate [195]. These pollutants can come from both natural sources, such as wildfires and volcanic eruptions, and human activities, such as industrial processes, transportation, and energy production. Developing sensors for air pollution is crucial because it empowers us to monitor and understand the quality of the air we breathe, enabling timely interventions to protect public health and the environment. Various types of sensors are being used in the development of devices for air pollution.

Rioual et al. [196] developed low-cost and low-visual-nuisance air-quality sensors based on radio-frequency identification (RFID) technology by utilizing reactive metallic dosimeters, which can be easily integrated into existing RFID applications and provide chemical analysis to determine pollutant origins. Pillarisetti et al. [197] developed a suite of affordable and durable microchip-based devices for quantifying parameters related to household air pollution, including particle monitors, temperature sensors, time–activity monitoring systems, and a CO_2_-based tracer-decay system, which have been extensively used in global studies to improve our understanding of household air pollution concentrations and exposures. Zikova et al. [198] used low-cost sensors to measure ambient particulate matter (PM) in multiple locations, revealing moderate spatial inhomogeneity and coherent variation driven by meteorological conditions and PM transport. Despite limitations in accuracy, these sensors proved useful for assessing spatial and temporal variations in PM concentrations and providing estimates based on central site monitoring data. Broday et al. [199] conducted comprehensive studies on the practical implementation of Wireless Distributed Environmental Sensor Networks for air-quality monitoring, assessing their capability to capture fine spatiotemporal urban pollutant patterns; the findings revealed the challenges of sensor interference and the need for frequent calibrations, highlighting the importance of developing suitable field calibration procedures to ensure reliable data for assessing the effects of air pollution on public health and human well-being. Caubel et al. [200] developed the Aerosol Black Carbon Detector (ABCD), a low-cost sensor designed to measure black carbon (BC) in densely distributed wireless networks, demonstrating its comparable measurement performance to a commercial BC instrument and presenting a data processing methodology that improves measurement accuracy in unconditioned operating environments, thus filling a critical gap in low-cost air pollution sensing technology.

Lastly, Ye et al. [190] developed a flexible La${}_{0.7}$Sr${}_{0.3}$MnO${}_{3}$ (LSMO)/Mica respiration sensor integrated into a face mask, enabling effective human breath monitoring with multi-modal capabilities and stability. It demonstrated the ability to discern different breath statuses (cough, normal breath, deep breath) and paves the way for novel flexible and wearable electronic devices in personal and public health applications. See Figure 8C.

#### 3.2.5. Deforestation

**Figure 9:**
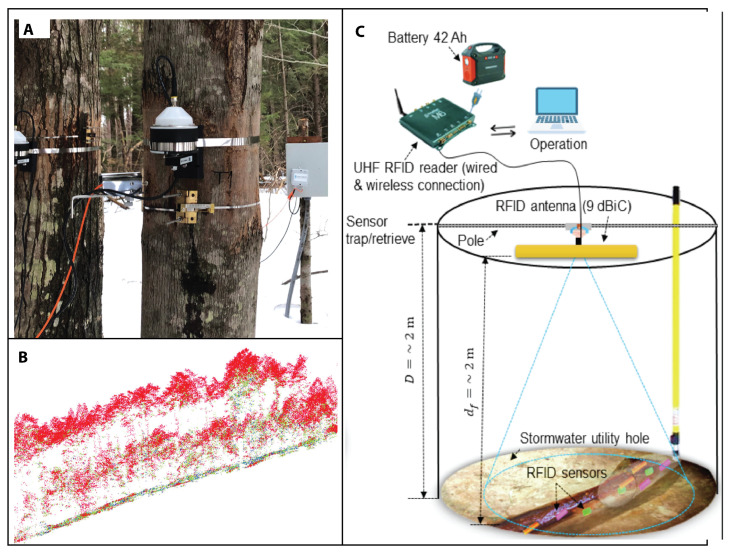
(**A**) Climate-smart forestry utilizes a comprehensive network of sensors interconnected through IoT to monitor forest conditions in real time, providing early warning signals of ecosystem regime shifts. (**B**) Light Detection and Ranging (LiDAR) and Radio Detection and Ranging (RADAR) technologies enable the highly accurate detection of vegetation canopy components and subcanopy topography, as demonstrated by a LiDAR system’s point cloud slice. The figure shows a slice of a point cloud acquired by a LiDAR system, with points colored by return number (red, first return; green, second return; blue, third return), evidencing the two-layered structure of a forest. Reprinted (adapted) with permission from [202]. (**C**) Flushable ultrahigh-frequency (UHF)-RFID-based sensors, address sewer blockages and illicit connections, offering an easy-to-operate, high-throughput, accurate, and low-cost solution. Field trials in Auckland, New Zealand, validated the feasibility of UHF-RFID sensors for digital water management. Adapted with permission from referenced sources [206].

Deforestation, the removal of trees and vegetation, is a critical environmental issue due to its impact on climate regulation and biodiversity. Trees absorb carbon dioxide and release oxygen, mitigating climate change effects. They also provide habitats for numerous species. Deforestation has social and economic consequences [201]. Sensors offer promising solutions for forest monitoring and management. Microdevices and nanotechnology are being implemented to create sensors that can monitor various environmental factors, such as temperature, humidity, and soil moisture. See Figure 9A,B. This information can be used to detect changes in the forest ecosystem and to identify areas that may be at risk of deforestation and to detect early warning signals of ecosystem regime shifts in terms of forest productivity, health, and biodiversity [202].

Mutiara et al. [203] proposed an integrated system utilizing microcontrollers, a sound sensor, and an accelerometer sensor, among other components, to monitor and track the positions of cut logs in forests. They demonstrated the real-time detection of illegal logging and achieved a tracking performance with tracking times ranging from 5 to 46 s for new log positions. Similarly, Prasetyo et al. [204] developed a prototype system utilizing a combination of a sound sensor and vibration sensor, integrated with a microcontroller and a Global System for Mobile (GSM) module, to detect illegal logging activities by identifying chainsaw sounds and detecting tree-falling vibrations, with optimized threshold values of 63.4 dB and 4400, respectively, providing an effective technology-based approach for reducing the occurrence of illegal logging. Andreadis et al. [205] proposed and evaluated a framework for automatically detecting illegal tree-cutting activity in forests using audio event classification by employing tiny ultra-low-power devices with edge-computing microcontrollers and long-range wireless communication and utilizing an efficient and accurate audio classification solution based on convolutional neural networks for resource-constrained wireless edge devices, achieving an accuracy of 85% in detecting tree-cutting events for cost-effective forest monitoring through smart IoT.

#### 3.2.6. Fatbergs

Fatbergs, comprising congealed fat, oil, grease (FOG), and non-biodegradable materials such as wipes and sanitary products, pose a significant threat to sewer systems, leading to costly blockages. Implementing sensors becomes crucial in the early detection, monitoring, and prevention of fatberg formation, effectively mitigating issues such as blockages, sewer corrosion, odors, and environmental pollution. This sensor-based approach enables the efficient maintenance and preservation of sewer infrastructure, facilitating the development of intelligent sewer systems. Tatiparthi et al. [206] developed flushable ultrahigh-frequency radio-frequency identification (UHF RFID) sensors for quick surveys of sanitary and storm-water pipes, enabling the low-cost, high-throughput, and robust monitoring of blockages, illicit connections, and water flow in sewer networks to prevent the release of pathogens into the environment, overcoming the limitations of current technologies. See Figure 9C.

## 4. Discussion

This study presents some of the most significant progress in sensors in the biomedical and environmental fields. These technologies have enabled unprecedented sensitivity and new ways of detecting analytes and events.

In the biomedical field, sensors have been enabled disease detection and diagnosis, drug discovery, and point-of-care devices. Electrochemical biosensors, for example, have been used to detect biomolecules such as glucose, lactate, and cholesterol for diagnosing diabetes and cardiovascular diseases. The use of sensors in drug discovery and antibiotic resistance research has also been significant, particularly those integrating microfluidics. These devices can screen a large number of drug candidates and characterize their biological activities with high accuracy and throughput, enabling the development of new drugs and therapies. Furthermore, the integration of sensors with artificial intelligence and machine learning has enabled further insight into the data and expanded potential applications.

In the environmental field, sensors have been used to monitor air, water, pollution, and food safety. These devices have been shown to detect pollutants with high accuracy and in real time, which may enable timely interventions to mitigate environmental pollution or provide insight into industrial activities. In recent years, biosensors that use biological components to detect environmental pollutants have been developed, enabling the high-sensitivity and high-selectivity detection of pollutants.

While the biomedical and environmental applications of sensors may seem different at first glance, they share several fundamental technologies that enable them to accurately and reliably measure physical, chemical, or biological phenomena. These technologies include transducer elements, signal processing, data communication, data analytics (statistical modeling, machine learning), and power management. As a result, advances and obstacles can be readily exchanged between these two fields, leading to a deeper understanding of and greater progress in the development and utilization of sensor technologies. Despite the significant progress made in sensors, several challenges remain to be addressed. For instance, there is a need to develop sensors that can detect a wider range of analytes with higher sensitivity and selectivity. Some progress has been made in the simultaneous detection of multiple analytes using a single sensor and multiplexed sensors; these sensors can be used for high-throughput measurements, reducing the cost and time required for analysis. Moreover, the integration of sensors with other technologies, such as wireless communication to enable IoT, has significantly progressed over the last years. In addition, the challenge of power management is opening the door for research opportunities in energy harvesting. Another significant challenge in the design and fabrication of these devices is the development of automated methods that allow reliability and consistency to be maintained outside of the laboratory using similar fabrication techniques. Additionally, there is a need to develop standards and regulations to ensure the safety and efficacy of these devices.

## 5. Perspective

Recent progress in the development of sensors has demonstrated their immense potential in addressing critical challenges in both biomedical and environmental domains. To fully leverage the capabilities of these sensors into devices, it is imperative to focus on the specific biomedical and environmental challenges identified, including infectious diseases, noncommunicable diseases, antibiotic resistance, the aging population, plastic pollution, eutrophication, deforestation, air pollution, climate change, and fatberg management and prevention.

In the realm of biomedical challenges, the design of sensors and devices should prioritize simplifying sample preparation procedures. Streamlining the sample preparation process not only saves time but also enhances the feasibility of point-of-care applications, allowing for rapid disease diagnosis and monitoring. Additionally, identifying suitable materials for sensor production is crucial to ensure both laboratory and commercial scalability. These materials should possess the necessary properties for sensor fabrication while also meeting safety and regulatory requirements. Furthermore, an important consideration in sensor design and characterization is the implementation of automated procedures, from the sensor layout design and fabrication to the characterization of the sensor. Automation not only improves operational efficiency but also enables sensor characterization in real time, offering valuable insights for disease management and drug discovery.

In the environmental domain, sensors can play a vital role in addressing challenges such as plastic pollution, eutrophication, deforestation, air pollution, climate change, and fatberg management and prevention. The design of these sensors should take into account the unique requirements of each challenge, including the ability to detect and monitor pollutants with high sensitivity and specificity. Moreover, simplifying the procedures for sample preparation is equally important in the environmental context, allowing for efficient monitoring and timely interventions to mitigate environmental damage. Additionally, sensor design should incorporate automated processes to streamline the operation of sensor design and characterization, facilitating real-time data collection and analysis for effective decision making and interventions.

Addressing these challenges and considerations in the design of sensors is pivotal to their successful adoption in biomedical and environmental applications. By simplifying sample preparation procedures, identifying suitable materials for sensor production, and incorporating automation, these sensors/devices can unlock their full potential in revolutionizing disease diagnosis, monitoring, and environmental management. Furthermore, collaboration between researchers, engineers, and stakeholders is crucial to drive innovation and ensure the translation of these advancements from the laboratory to commercial settings.

## 6. Conclusions

In conclusion, the recent progress in the development of sensors has demonstrated their significant potential in addressing critical challenges in both the biomedical and environmental fields. These devices have revolutionized the detection and quantification of various analytes, enabling advancements in disease diagnosis, drug discovery, environmental monitoring, and food safety. Integration with emerging technologies, such as artificial intelligence and machine learning, has further expanded their potential applications, paving the way for novel solutions in healthcare and environmental sustainability.

While significant strides have been made, several challenges remain to be addressed to fully leverage the capabilities of sensors. One key challenge is the development of sensors capable of detecting a wider range of analytes with higher sensitivity and selectivity. Advancements in multiplexed sensors offer promise in simultaneously detecting multiple analytes, reducing the time and cost of analysis. Additionally, the integration of sensors with emerging technologies, such as wireless communication and energy harvesting, presents opportunities for enhanced capabilities and autonomous operation.

To capitalize on the potential of sensors, it is crucial to address the specific challenges identified in the biomedical and environmental domains. These challenges encompass infectious diseases, noncommunicable diseases, antibiotic resistance, the aging population, plastic pollution, eutrophication, deforestation, air pollution, climate change, and fatberg management and prevention. Simplifying sample preparation procedures, identifying appropriate sensor production materials, and incorporating automation into sensor design and characterization are vital steps toward widespread adoption and deployment.

By emphasizing these considerations, we can enhance disease diagnosis, monitoring, and management while effectively addressing pressing environmental concerns. Collaboration among researchers, engineers, and stakeholders is essential to drive innovation and ensure the successful translation of advancements from the laboratory to commercial settings. With a focus on simplification, automation, and integration, sensors have the potential to usher in a healthier and more sustainable future for all, where critical challenges are effectively tackled and improved outcomes are realized in both biomedical and environmental contexts.

## Data Availability

Not applicable.

## Abbreviations

The following abbreviations are used in this manuscript:

AgNPs	Ag nanoparticles
AD	Analytical devices
AST	Antibiotic susceptibility testing
BNP	Brain natriuretic peptide
CO	Carbon monoxide
cTnI	Cardiac troponin I
CVDs	Cardiovascular diseases
CCDs	Charge-coupled devices
CSF	Climate-smart forestry
CPW	Coplanar waveguide
PCNN	Coupled neural network
EWS	Early warning signals
ECG	Electrocardiogram
ECL	Electrochemiluminescence
EEG	Electroencephalogram
EMG	Electromyogram
ePADs	Electrochemical paper-based analytical devices
EPS	Extracellular polymeric substances
FOG	Fat, oil, and grease
FET	Field-effect transistor
GFET	Graphene field-effect transistor
GQDs	Graphene quantum dots
GSM	Global System for Mobile
IoT	Internet of Things
LAMP	Loop-mediated isothermal amplification
LiDAR	Light Detection and Ranging
LOD	Limit of detection
LSPR	Localized surface plasmon resonance
MDPI	Multidisciplinary Digital Publishing Institute
MFCs	Microbial fuel cells
MEMS	Microelectromechanical systems
MPs	Microplastics
MSCs	Microsupercapacitors
MWNTs	Multiwalled carbon nanotubes
MTZ	Metronidazole
NC	Nanocrystals
NEFET	Nanopore extended field-effect transistor
BNP-32	Natriuretic peptide
NOx	Nitrogen oxides
NCAs	Nitrogen-doped carbon aerogels
NCD	Noncommunicable diseases
OoC	Organ-on-a-chip
ORR	Oxygen reduction reaction
PBMC	Peripheral blood mononuclear cell
PDs	Photodetector
PO	Plasmonic oxide
POC	Point of care
PDMS	Polydimethylsiloxane
PEMC	Porous Ecoflex-multiwalled carbon nanotube composite
PA	Precision agriculture PA
QDs	Quantum dots
QCM-D	Quartz crystal microbalance dissipation
RADAR	Radio Detection and Ranging
RF	Radio frequency
RFID	Radio-frequency identification
RIU	Refractive Index Units
SAW	Surface acoustic wave
SSPP	Spoof surface plasmon polaritons
SO_2_	Sulfur dioxide
SPPs	Surface plasmon polaritons
SPR	Surface plasmon resonance
SRR	Split-ring resonator
SERS	Surface-enhanced Raman spectroscopy
THz	Terahertz
TPTS	Triboelectric palm-like tactile sensor
TEGs	Thermoelectric generators
VOCs	Volatile organic compounds
